# Plant Litter Submergence Affects the Water Quality of a Constructed Wetland

**DOI:** 10.1371/journal.pone.0171019

**Published:** 2017-01-27

**Authors:** Xu Pan, Yunmei Ping, Lijuan Cui, Wei Li, Xiaodong Zhang, Jian Zhou, Fei-Hai Yu, Andreas Prinzing

**Affiliations:** 1 Institute of Wetland Research, Chinese Academy of Forestry, Beijing, China; 2 Beijing Key Laboratory of Wetland Services and Restoration, Beijing, China; 3 Beijing Hanshiqiao National Wetland Ecosystem Research Station, Beijing, China; 4 School of Nature Conservation, Beijing Forestry University, Haidian District, Beijing, China; 5 Centre National de la Recherche Scientifique Campus de Beaulieu, Université de Rennes 1, Bâtiment 14 A, Rennes, France; Portland State University, UNITED STATES

## Abstract

Plant litter is an indispensable component of constructed wetlands, but how the submergence of plant litter affects their ecosystem functions and services, such as water purification, is still unclear. Moreover, it is also unclear whether the effects of plant litter submergence depend on other factors such as the duration of litter submergence, water source or litter species identity. Here we conducted a greenhouse experiment by submerging the litter of 7 wetland plant species into three types of water substrates and monitoring changes in water nutrient concentrations. Litter submergence affected water quality positively via decreasing the concentration of nitrate nitrogen and negatively via increasing the concentrations of total nitrogen, ammonium nitrogen and total phosphorus. The effects of litter submergence depended on the duration of litter submergence, the water source, the litter species identity, and the plant life form. Different plant species had different effects on the water nutrient concentrations during litter submergence, and the effects of floating plants might be more negative than that of emergent plants. These results are novel evidence of how the submergence of different plant (life form) litter may affect the purification function of constructed wetlands. For water at low eutrophication levels, submerging a relative small amount of plant litter might improve water quality, via benefiting the denitrification process in water. These findings emphasized the management of floating plant litter (a potential removal) during the maintenance of human-controlled wetland ecosystems and provided a potential tool to improve the water quality of constructed wetlands via submerging plant litter of different types.

## Introduction

Wetland plant species are important components of constructed wetlands (CWs), which are engineered treatment systems that encompass a plurality of biological, chemical, and physical processes to improve water quality [1]. A wide range of waste waters from various origins, such as domestic, industrial, agricultural and even landfill leachate, can be treated by CWs [2–5]. Globally, the interest in using the purification function of CWs to improve the quality of polluted or nutrient rich water is increasing [6] and the important role of wetland plants in improving water quality has been recognized. For example, wetland plant species such as emergent plants can support sedimentation, prevent re-suspension and provide substrate for microorganisms and algae [1]. Moreover, wetland plants can also assimilate nutrients, create favorable conditions for the microbial decomposition of organic matter and provide carbon sources for denitrification to improve water quality in constructed wetlands [7–10].

Plant litter might play an important role in affecting the water quality of CWs. For example, plant litter via decomposition processes may release substantial nutrients, benefit plant growth [11], promote the abundance and diversity of the microbial community or significantly change microbial community compositions at the class and genus levels [12], and benefit the denitrification processes in carbon-limited wetland conditions [6, 9], improving the water quality of CWs. On the other hand, plant litter decomposition might lead to eutrophication in CWs [13] and the litter of wetland plants, especially emergent plants, during senescence is commonly removed from CWs. Therefore, it is still unclear what role the litter of wetland plant species may play in affecting water quality and whether such a role might depend on the nutrient condition of the water sources.

A few studies have shown that plant species differ greatly in their ability to purify water in wetlands [14, 15] and wetland plant species are very specific in their ability to uptake nutrients [8]. Based on a literature review of 643 CWs, there are over 150 macrophyte species used in CWs worldwide [9], and among those species, the most commonly used species are *Typha* spp., *Scirpus* spp., *Phragmites* spp., *Juncus* spp. and *Eleocharis* spp. [9]. Wetlands constructed with *Typha* spp., for instance, show a higher ability to remove nitrogen than those constructed with *Scirpus* spp. [14], and mesocosms with *Phragmites* spp. and *Canna* spp. were more efficient at removing contaminants than those with other wetland plant species [16]. Differences among species may reflect the species’ life form [8] and/or litter quality. Litter quality differs greatly among plant species [17] and to some degree these differences reflect variations in plant functional traits as the plant only resorbs part of its nutrients during leaf senescence [17–19]. However, the effects of plant species litter on water quality is still insufficiently known and it is also unknown whether these effects might depend on plant life form.

We carried out a greenhouse experiment to examine the effects of plant litter submergence on the water nutrient concentrations, such as the concentrations of nitrate nitrogen (NO_3_-N), ammonium nitrogen (NH_4_-N), total nitrogen (TN) and total phosphorus (TP). We tested with litter of 7 wetland plant species of two life forms (floating vs. emergent plants) and three different water sources. We addressed three questions: (1) How does litter submergence affect water nutrient concentrations through time, and (2) whether is there a similar pattern of changes for different water nutrients during litter submergence? (3) Do the effects of litter submergence depend on plant species identity and/or plant life forms?

## Materials and Methods

### The constructed wetland

Our study site is located in the Beijing Wildlife Rescue and Rehabilitation center in Shunyi district, Beijing, China (Latitude: 40°6ʹ14.40ʺN, Longitude: 116°42ʹ35.71ʺE). This center is a protected area and specific permission should be issued by Beijing Municipal Bureau of Landscape and Forestry ahead of time. In this center, there was a constructed wetland designed by the Institute of Wetland Research, Chinese Academy of Forestry. This constructed wetland belongs to an integrated constructed wetland, which was used to improve the water quality of an artificial lake at the site (S1 Fig; the detailed design of the constructed wetland can also be seen in [20]). The CW was divided into 12 sections, which were named from A to L (A-I belongs to the surface flow constructed wetland; J-L belongs to the subsurface flow constructed wetland), and the water in the artificial lake which is polluted by the water bird sewage flows into the CW from the section A and goes back to the lake from the section L. Within each section, different wetland plant species, such as *P*. *australis*, *T*. *orientalis* and other wetland plant species, were planted to purify polluted water from the artificial lake.

### Experimental design

We carried out a greenhouse experiment to examine the influence of plant litter submergence on the water nutrient concentrations of a constructed wetland. Using 72 plastic boxes (40 cm length, 30 cm width and 15 cm height), we divided them into three groups and filled with water of different water sources: the first group was filled with water-fowl polluted water (water A), which was collected at the start of the constructed wetland (section A); the second group was filled with purified water collected at the end of the surface-flow section of the same construct wetland (section I) (water B); the third group was filled with the local treated tap water (water C). All the water was collected on the same day.

We also collected fresh litter of 7 wetland plant species growing in the constructed wetland (S1 Table), including 2 floating plants (*Salvinia natans* and *Lemna minor*) and 5 emergent plants (*Iris wilsonii*, *Zizania latifolia*, *Sparganium stoloniferum*, *Typha orientalis* and *Phragmites australis*). These species are all considered helpful in contributing to the purification of water in constructed wetlands [20]. All these plant litters were air dried in the greenhouse, then we prepared 9 litter bags for each litter species. For floating plant species, we weighed around 20 g litter for each litter bag; but for the emergent plant species, we weighed around 50 g litter for each litter bag. All these litter bags were then immersed into the plastic boxes. Each plastic box contained only one litter type and there were in total of 63 boxes which contained litter bags, and the other 9 boxes were the control treatment with no litter submergence. The number of replicates was three throughout the experiment. All the boxes were covered with insurance membrane in order to decrease evaporation of water and also decrease the gas exchange between the water and the air. We changed insurance membrane every two weeks. This whole experiment last from October 28, 2015 to December 24, 2015 with the duration of 57 days. Changes of water quality in the plastic boxes were monitored after 2 weeks, 4 weeks, 6 weeks and 8 weeks.

### Measurements of water and litter qualities

We measured water nutrient concentrations both before and after litter submergence. For nutrient analysis, we used a Smart-Chem spectrophotometer (WESTCO Scientific Instruments, Brookfield, CT, USA) to measure the concentrations of total nitrogen (TN), total phosphorus (TP), nitrite nitrogen (NO_3_-N) and ammonium nitrogen (NH_4_-N). Moreover, the total carbon and total nitrogen concentrations of the litter were analyzed by an automated elemental analyzer. P concentrations in litter were analyzed by inductively coupled plasma emission spectroscopy (Perkin Elmer Optima 3000 ICP Spectrometer, Waltham, MA). In addition, we used a multi-parametric probe (YSI 6820, YSI Environmental Inc., USA) to measure water temperature, dissolved oxygen, electrical conductivity and total dissolved solids during litter submergence.

### Statistical analysis

All data were checked for the assumptions of homogeneity of variance and normality before analysis. We log-transformed the data which did not fit those assumptions. Then we analyzed the effects of measurement time (T), initial water sources (S) and litter species (L) on water nutrient concentrations mentioned above using repeated measures ANOVA in SPSS Statistics (SPSS, Chicago, IL, USA). Moreover, we replaced the litter species with plant life forms (E: emergent plants; F: floating plants; CK: control treatment with no litter submergence) and did another repeated measures ANOVA to test the effects of plant life forms (PF), time of litter submergence and initial water sources on water nutrient concentrations. Differences between means were tested with Turkey tests and orthogonal comparisons; effects were considered significant at *p* < 0.05. In addition, we did similar analyses for water temperature, dissolved oxygen, electrical conductivity and total dissolved solids. These statistical results were shown in the Appendix (S2 Table).

## Results

**Fig 1:**
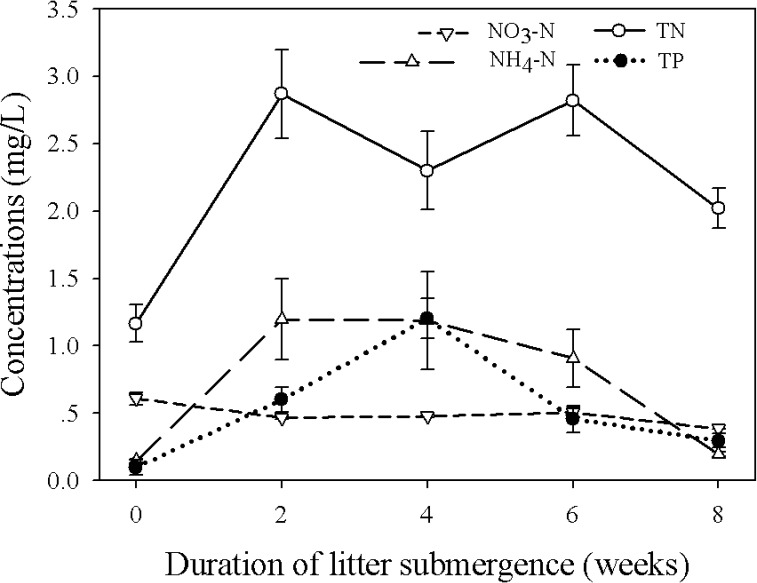
Changes in water nutrient concentrations through time during litter submergence, including nitrate nitrogen (NO_3_-N), ammonium nitrogen (NH_4_-N), total nitrogen (TN) and total phosphorus (TP). The white circles and the solid line represent the changes in TN; the positive triangles and the long-dashed line represent the changes in NH_4_-N; the inverted triangles and short-dashed line represent the changes in NO_3_-N; the black circles and the dot line represent the changes in TP.

Litter submergence had significant effects on water nutrient concentrations through time: TN concentration (Table 1: *F*_3,144_ = 7.44 and *F*_3,189_ = 7.39, *p* < 0.01), TP concentration (Table 1: *F*_3,144_ = 34.02 and *F*_3,189_ = 16.50, *p* < 0.01), NO_3_-N concentration (Table 1: *F*_3,144_ = 9.26 and *F*_3,189_ = 5.27, *p* < 0.01) and NH_4_-N concentration (Table 1: *F*_3,144_ = 42.85 and *F*_3,189_ = 13.84, *p* < 0.01). For nitrogen, litter submergence significantly increased water total N and NH_4_-N, and the concentrations of total N and NH_4_-N were the highest after 2 weeks (Fig 1). However, litter submergence decreased the NO_3_-N concentration through time (Fig 1). For total P concentration, litter submergence also increased the P concentration and the highest concentration was seen after 4 weeks (Fig 1).

**Table 1:** Repeated measures ANOVA results for the effects of measurement time (2 weeks, 4 weeks, 6 weeks and 8 weeks), initial water sources (polluted water, purified water and tap water) and litter species or plant life forms on water nutrient concentrations, such as nitrate nitrogen (NO_3_-N), ammonium nitrogen (NH_4_-N), total nitrogen (TN), and total phosphorus (TP).

	Variables			
	NO_3_-N	NH_4_-N	TN	TP
*Effects of litter species and water substrate*				
*Between subject Effects*				
Water sources (S)	F_2,48_ = 5.54**	F_2,48_ = 5.72**	F_2,48_ = 1.41^ns^	F_2,48_ = 1.12^ns^
Litter species (L)	F_7,48_ = 6.04**	F_7,48_ = 52.90**	F_7,48_ = 41.46**	F_7,48_ = 26.68**
S * L	F_14,48_ = 4.49**	F_14,48_ = 5.48**	F_14,48_ = 4.50**	F_14,48_ = 0.63^ns^
*Within subject Effects*				
Measurement time (T)	F_3,144_ = 9.26**	F_3,144_ = 42.85**	F_3,144_ = 7.44**	F_3,144_ = 34.02**
T * S	F_6,144_ = 1.49^ns^	F_6,144_ = 4.06**	F_6,144_ = 4.74**	F_6,144_ = 1.10^ns^
T * L	F_21,144_ = 3.72**	F_21,144_ = 36.86**	F_21,144_ = 2.70**	F_21,144_ = 3.93**
T * S * L	F_42,144_ = 2.65**	F_42,144_ = 3.00**	F_42,144_ = 1.38^ns^	F_42,144_ = 1.59*
*Effects of plant life form and water substrate*				
*Between subject Effects*				
Water sources (S)	F_2,63_ = 5.86**	F_2,63_ = 1.47^ns^	F_2,63_ = 1.01^ns^	F_2,63_ = 0.20^ns^
Plant life form (PF)	F_2,63_ = 17.67**	F_2,63_ = 16.51**	F_2,63_ = 13.10**	F_2,63_ = 5.07**
S * PF	F_4,63_ = 8.77**	F_4,63_ = 1.93^ns^	F_4,63_ = 1.39^ns^	F_4,63_ = 0.14^ns^
*Within subject Effects*				
Measurement time (T)	F_3,189_ = 5.27**	F_3,189_ = 13.88**	F_3,189_ = 7.39**	F_3,189_ = 16.50**
T * S	F_6,189_ = 1.01^ns^	F_6,189_ = 1.30**	F_6,189_ = 5.24**	F_6,189_ = 0.84^ns^
T * PF	F_6,189_ = 2.07^ns^	F_6,189_ = 16.62**	F_6,189_ = 4.97**	F_6,189_ = 5.13**
T * S * PF	F_12,189_ = 1.63^ns^	F_12,189_ = 1.44^ns^	F_12,189_ = 2.12^ns^	F_12,189_ = 1.11^ns^

Note

** represents the significance at *p* < 0.01

* represents the significance at the level of *p* < 0.05; ns represents no significant differences, i.e. *p* > 0.05.

**Fig 2:**
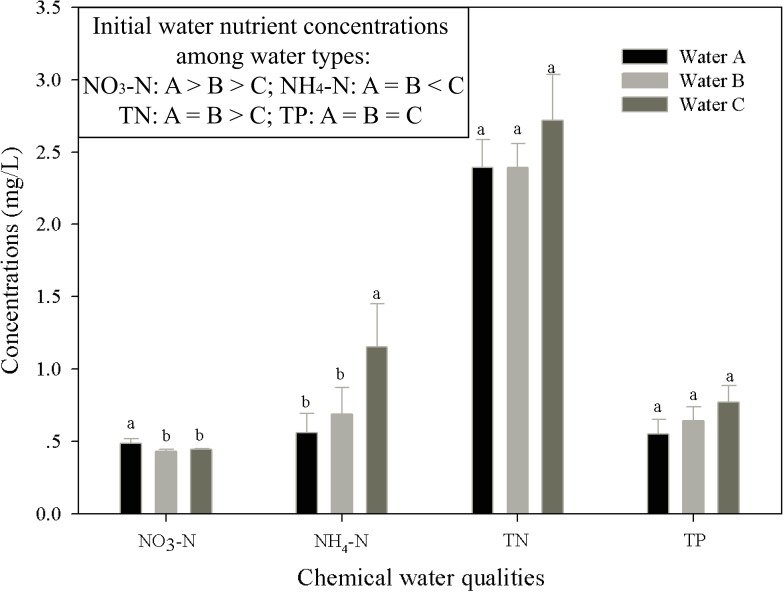
**Effects of different water sources (water A: polluted water with water-fowl sewage; water B: purified water collected at the end of the surface-flow section of a constructed wetland; water C: local tap water) on water nutrient concentrations during litter submergence, including nitrate nitrogen (NO**_**3**_**-N), ammonium nitrogen (NH**_**4**_**-N), total nitrogen (TN) and total phosphorus (TP).** Bars show the standard error (SE).

The initial nutrient concentrations of the three water sources differed significantly: polluted water (A) had the highest NO_3_-N, followed by the purified water (B) and the tap water (C), but the tap water had higher N and lower NH_4_-N than the polluted water and the purified water, both of which had similar concentrations of N and NH_4_-N (Fig 2). There were no significant differences in the P concentrations of the initial water sources. During litter submergence, the substrate water types led to significant differences in NO_3_-N and NH_4_-N concentrations (Table 1, NO_3_-N: *F*_2,48_ = 5.54 and *F*_2,63_ = 5.86, *p* < 0.01; NH_4_-N: *F*_2,48_ = 5.72 and *F*_2,63_ = 1.47; *p* < 0.01 and *p* = 0.24), but not in N and P concentrations (Table 1, TN: *F*_2,48_ = 1.41 and *F*_2,63_ = 1.01, *p* > 0.05; TP: *F*_2,48_ = 1.12 and *F*_2,63_ = 0.20; *p* > 0.05).

**Fig 3:**
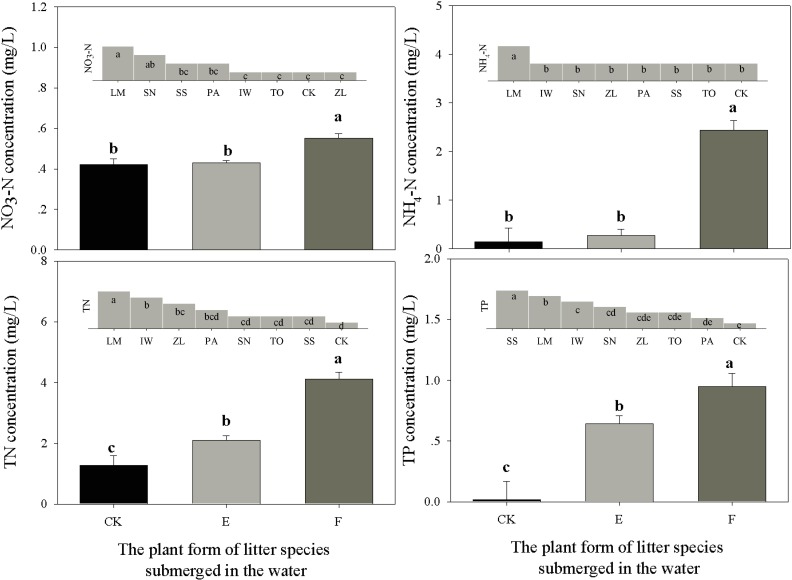
Effects of plant growth forms (E: emergent plants; F: floating plants; CK: control with no litter submergence) and litter species (SN: *Salvinia natans*; LM: *Lemna minor*; IW: *Iris wilsonii*; ZL: *Zizania latifolia*; SS: *Sparganium stoloniferum*; TO: *Typha orientalis*; PA: *Phragmites australis*; CK: control with no litter submergence) on water nutrient concentrations during litter submergence. Nutrients included nitrate nitrogen (NO_3_-N), ammonium nitrogen (NH_4_-N), total nitrogen (TN) and total phosphorus (TP). Effects of different species on nutrient concentrations, which were grouped into 2–7 levels based on the multiple comparison results, were ranked from the largest to the smallest. The lower case letters (a-f) represent the results of multiple comparisons among litter species. Bars show the standard error (SE).

**Fig 4:**
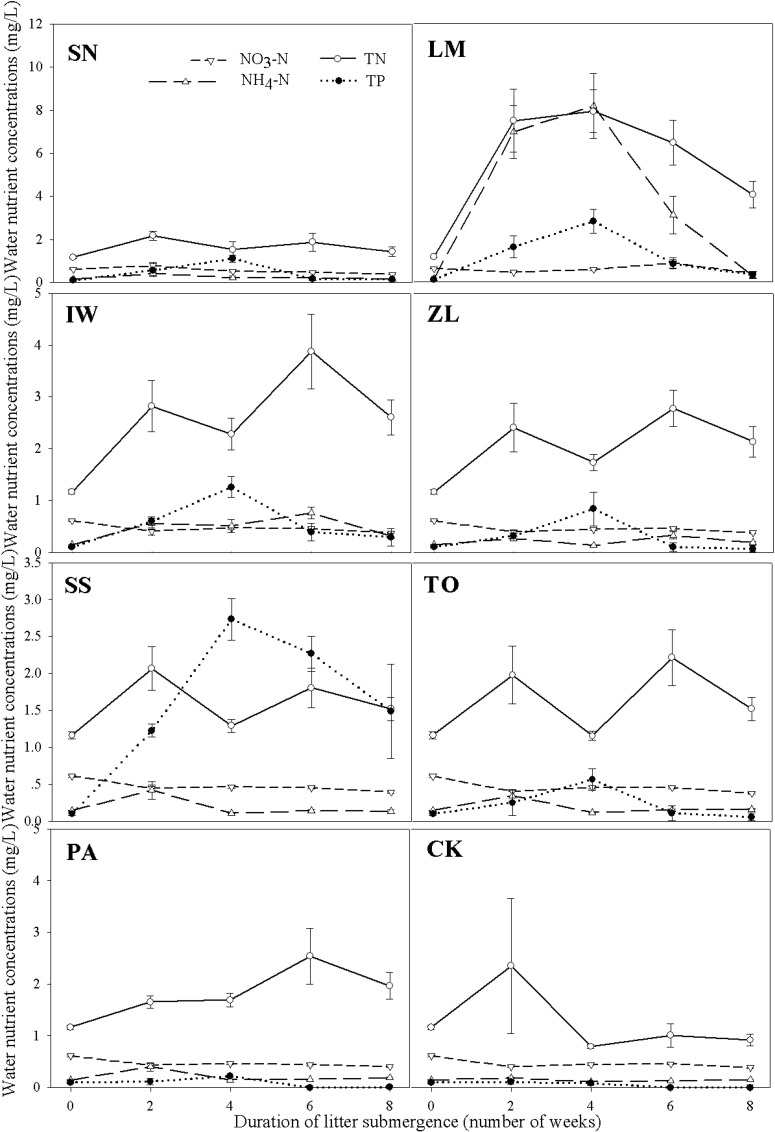
Changes in water nutrient concentrations among different wetland species during litter submergence: nitrate nitrogen (NO_3_-N), ammonium nitrogen (NH_4_-N), total nitrogen (TN) and total phosphorus (TP). The white circles and the solid line represent the changes in TN; the positive triangles and the long-dashed line represent the changes in NH_4_-N; the inverted triangles and short-dashed line represent the changes in NO_3_-N; the black circles and the dot line represent the changes in TP. Bars show the standard error (SE).

The growth forms of wetland plant species had significant effects on water nutrient concentrations (Table 1, Fig 3). The concentrations of all water nutrients were the highest in mesocosms with litter from floating plants, followed by the litter of emergent plants (Fig 3). The litter of emergent plants led to higher N and P concentrations than the control treatment (no litter), but led to no difference in NO_3_-N and NH_4_-N concentrations (Fig 3). As to the effect of litter species, litter of *L*. *minor* led to the highest concentrations of N, NO_3_-N and NH_4_-N, and the second highest concentration of P; litter of *T*. *orientalis*, *P*. *australis* and *Z*. *latifolia* led to similar water qualities as the control treatment (Fig 3); litter of *S*. *stoloniferum* led to significantly higher concentration of P than the other species (Fig 4). Moreover, litter species had significant main effects and also significant interaction effects with measurement time on water nutrient concentrations (Table 1, Fig 4).

## Discussion

Plant litter is an important source of nutrients in constructed wetlands, and large amounts of nutrients released from plant litter may lead to eutrophication, change the species composition of wetland vegetation and cause other biological changes [6, 21, 22]. The effects of litter submergence on water nutrient concentrations depended on the duration of litter submergence, initial water sources as well as plant species identity and/or plant life form (Table 1).

In this study plant litter might promote the water eutrophication in a short period. Litter submergence increased the concentrations of TN, TP and NH_4_-N in water through time, although those concentrations increased at the first 4 weeks and then decreased during the following 4 weeks (Fig 1). Plant litter might leach more N and P to the water under longer litter submergence than shorter litter submergence [22]. Moreover, the litter, as a carbon source for microbes, may promote some geochemical processes such as the denitrification [10, 23–25]. This promotion was proven to happen when the concentration of nitrate is less than 50 mg/L [10]. We observed this promotion in our study, that NO_3_-N decreased throughout the whole experiment, implying that plant litter submergence might promote nitrate removal from the water possibly via denitrification processes. Therefore, these results suggested that plant litter might have both negative and positive effects on water quality in CWs, although the positive effect was weak. The direction of the litter effects might depend on the initial concentration of nutrients, or the characteristics of plant litter.

We found that plant litter played different roles in affecting water quality depending on the initial water sources. The water collected from the constructed wetland (water A and B, higher initial N concentration, Fig 2) and the natural tap water (water C) showed different trends in nutrient concentration changes with litter submergence. The concentrations of TN and NH_4_-N in water A and B were lower than that in water C, and the concentrations of NO_3_-N decreased more in water A and B than that in water C. Our finding showed opposite results with previous studies, which indicated that the streams or water bodies with higher nutrient concentrations (N and P) might lead to faster litter decomposition rates [26–29], implying more nutrient released into the water. The lower TN and NH_4_-N concentrations and the slower NO_3_-N concentration decrease in water A and B might attribute to the micro-organisms activities, which were more abundant in the water collected from the constructed wetland. Since the water bodies in our study system were static with lower oxygen content, where the aerobic process such as mineralization and nitrification might get slower, while the anaerobic process such as denitrification get faster.

We also found that the effects of litter submergence on water nutrients also differed significantly among litter plant species and plant life forms (Table 1, Fig 3), probably via different rates of mass losses and nutrient release (S3 Table, mass losses ranged from 20% to 80% and the nutrient losses varied even more among species). Wetland plant species may differ significantly in plant functional traits, which might have different consequences for ecosystem functioning [17, 26, 30–32]. The litter decomposition of floating plants might be faster than that of emergent plants, as they have softer and more decomposable materials [33]. The floating plant litter used in this study had lower C/N ratios (F vs. E: 11.55 vs. 30.70) and higher TN concentration (F vs. E: 3.66% vs. 1.88%) than emergent plant litter, leading to faster decomposition and nutrient release into the water. Meanwhile, the emergent plant litter with higher C/N ratio may promote the denitrification process, as the previously study suggested that the denitrification rate might depend on the C/N ratio of the water and the carbon sources during incubation [10]. Moreover, other leaf or litter traits, such as leaf area and litter toughness [26, 27,33], might also contribute to the observed differences between different plant life forms by affecting interactions among the litter surface, microbes and the water. However, this speculation needs to be tested in future.

Wetland plants used in CWs usually have the characteristics of rapid establishment, spread and growth [30], which will produce large amounts of litter. However, the produced litter is usually much more than what the CWs can process. Therefore, wetland managers usually remove plant litter directly to keep the CWs running. Our results have several implications for the management of CWs: (1) plant litter has both positive and negative effects on water quality, and wetland managers should wisely use plant litter in the management of CWs; (2) since the effects of litter submergence depended on the litter species identity, the actions avoiding the negative effects of litter submergence should be made based on the characteristics of different litter species; (3) the floating plant litter performed more negatively on the water quality than the emergent plant litter, thus the floating plant litter should be carefully managed in CWs.

Even though our study was a greenhouse experiment, our results have their applicability in constructed wetlands and/or other types of natural wetlands. For example, plant litter in natural wetlands was mostly in mixtures and hence the observed effects of litter submergence on water quality might be the overall effects of multiple species, but not at the species level. Our results revealed the effects of litter submergence on water quality at the species level, and this could help us to select appropriate plant species for constructed wetlands and better understand the role of different plant species in affecting wetland ecosystem functions and services. However, admittedly, this greenhouse experimental design could to some extent limit the applicability of our results. Our greenhouse experiment only explored the effect of litter submergence on static water with relatively low eutrophication, but multiple other factors in real wetland ecosystems might lead to different effects of litter submergence on water quality. Therefore, future research is needed to examine such effects in constructed wetlands or other types of natural wetlands.

## Conclusion

Most studies which examined the effect of litter decomposition on water quality usually examined the loss of litter mass and the release of litter nutrients during decomposition by monitoring the decomposed litter [22]. We chose to directly examine the interspecific differences in effects of litter submergence on water nutrients. We found that plant litter submergence could affect eutrophication both negatively and positively, leading to higher total N and P concentrations and lower NO_3_-N concentration in water. The role of litter in the CWs depended on the duration of litter submergence, water substrate quality, litter species identity and possibly the underlying plant functional traits or litter traits. In addition, other factors such as the amount of submerged litter and the pretreatment of the litter materials [10, 24] may also matter. We suggest that future studies focus on a longer period of litter submergence, involve in more plant species and try to explore more related plant functional traits or litter traits to connect litter submergence with water quality.

## Supporting Information

S1 Fig Location and schematics of the constructed wetland involved in the study. (PDF)

S1 Table Wetland plant litter species involved in this study. (PDF)

S2 Table Repeated measures ANOVA results for the effects of measurement time, initial water source and litter species or plant life forms on physical water qualities. (PDF)

S3 Table Mass losses, nutrient changes and losses of 7 wetland plant litter submerging in the water. (PDF)

S4 Table Main dataset for the analyses. (PDF)
