# Supplementary figures and images for: Plant Litter Submergence Affects the Water Quality of a Constructed Wetland
Source: PLoS One. 2017 Jan 27;12(1):e0171019. doi: 10.1371/journal.pone.0171019 (PMC5271387; doi:10.1371/journal.pone.0171019)

**S1 Fig. Location and schematics of the constructed wetland involved in the study.**

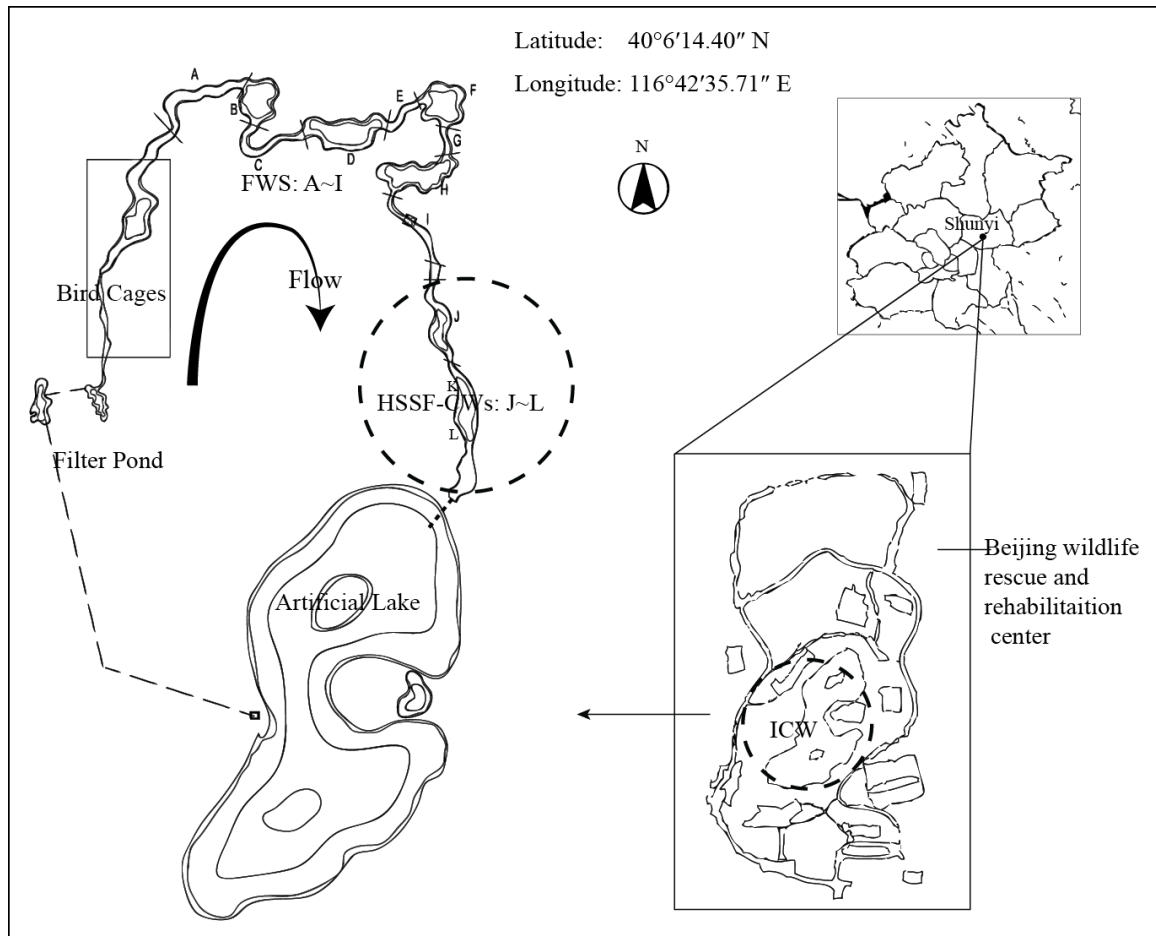

Supplement: S1 Fig — (PDF) [file pone.0171019.s001.pdf]
