# Supplementary material for: Plant Litter Submergence Affects the Water Quality of a Constructed Wetland
Source: PLoS One. 2017 Jan 27;12(1):e0171019. doi: 10.1371/journal.pone.0171019 (PMC5271387; doi:10.1371/journal.pone.0171019)
Supplement: S1 Table — (PDF) [file pone.0171019.s002.pdf]

S1 Table Wetland plant litter species involved in this study.

Species names	Life form (E or F)	Litter C (%)	Litter N (%)	C/N
Salvinia natans	F	39.10	2.55	8.20
Lemna minor	F	37.99	4.76	14.90
Iris wilsonii	E	43.49	0.95	45.79
Zizania latifolia	E	38.33	0.91	42.46
Sparganium stoloniferum	E	44.94	3.49	12.89
Typha orientalis	E	45.38	1.28	35.48
Phragmites australis	E	47.02	2.78	16.89
