# Supplementary material for: Plant Litter Submergence Affects the Water Quality of a Constructed Wetland
Source: PLoS One. 2017 Jan 27;12(1):e0171019. doi: 10.1371/journal.pone.0171019 (PMC5271387; doi:10.1371/journal.pone.0171019)
Supplement: S2 Table — (PDF) [file pone.0171019.s003.pdf]

S2 Table Repeated measures ANOVA results for the effects of measurement time (2 weeks, 4 weeks, 6 weeks and 8 weeks), initial water source (polluted water, purified water and tap water) and litter species or plant life forms on physical water qualities through time during litter submergence, including water temperature (Temp), water conductivity (Cond), total dissolved solids (TDS) and water dissolved oxygen (DO).

	Variables			
	DO	Cond	Temp	TDS
	Effects of litter species and water substrate			
Between subject Effects				
Substrate (S)	$F_{2,47}$ = 6.775 **	$F_{2,47}$ = 10.410 **	$F_{2,47}$ = 155.952 **	$F_{2,47}$ = 13.160 **
Litter species (L)	$F_{7,47}$ = 83.507 **	$F_{7,47}$ = 91.526 **	$F_{7,47}$ = 1.257 ns	$F_{7,47}$ = 79.468 **
S * L	$F_{14,47}$ = 4.855 **	$F_{14,47}$ = 4.078 **	$F_{14,47}$ = 2.315 *	$F_{14,47}$ = 3.592 **
Within subject Effects				
Measurement time (T)	$F_{3,141}$ = 330.17 **	$F_{3,141}$ = 415.2 **	$F_{3,141}$ = 4938.1 **	$F_{3,141}$ = 13.956 **
T * S	$F_{6,141}$ = 5.372 **	$F_{6,141}$ = 23.648 **	$F_{6,141}$ = 227.75 **	$F_{6,141}$ = 0.958 ns
T * L	$F_{21,141}$ = 14.75 **	$F_{21,141}$ = 13.49 **	$F_{21,141}$ = 7.907 **	$F_{21,141}$ = 10.92 **
T * S * L	$F_{42,141}$ = 1.953 **	$F_{42,141}$ = 1.292 ns	$F_{42,141}$ = 1.172 ns	$F_{42,141}$ = 1.073 ns
	Effects of plant life form and water substrate			
Between subject Effects				
Substrate (S)	$F_{2,62}$ = 0.191 ns	$F_{2,62}$ = 0.483 ns	$F_{2,62}$ = 86.266 **	$F_{2,62}$ = 0.692 ns
Plant life form (F)	$F_{2,62}$ = 62.239 **	$F_{2,62}$ = 48.497 **	$F_{2,62}$ = 2.084 ns	$F_{2,62}$ = 46.339 **

S * F	$F_{4,62}$ = 1.410 ns	$F_{4,62}$ = 0.525 ns	$F_{4,62}$ = 6.292 **	$F_{4,62}$ = 0.584 ns
Within subject Effects				
Measurement time (T)	$F_{3,186}$ = 84.32 **	$F_{3,186}$ = 132.6 **	$F_{3,186}$ = 2559.9 **	$F_{3,186}$ = 14.005 **
T * S	$F_{6,186}$ = 1.555 ns	$F_{6,186}$ = 8.168 **	$F_{6,186}$ = 106.58 **	$F_{6,186}$ = 0.501 ns
T * F	$F_{6,186}$ = 13.29 **	$F_{6,186}$ = 19.194 **	$F_{6,186}$ = 11.832 **	$F_{6,186}$ = 20.421 **
T * S * F	$F_{12,186}$ = 0.604 ns	$F_{12,186}$ = 0.613 ns	$F_{12,186}$ = 1.809 *	$F_{12,186}$ = 0.755 ns
