# Supplementary material for: Plant Litter Submergence Affects the Water Quality of a Constructed Wetland
Source: PLoS One. 2017 Jan 27;12(1):e0171019. doi: 10.1371/journal.pone.0171019 (PMC5271387; doi:10.1371/journal.pone.0171019)
Supplement: S3 Table — (PDF) [file pone.0171019.s004.pdf]

S3 Table Mass losses, nutrient changes and losses of 7 wetland plant litter submerging in the water.

Latin name	Massloss		N-change		P-change		N-loss		P-loss	
	Mean	SD	Mean	SD	Mean	SD	Mean	SD	Mean	SD
Salvinia natans	0.57	0.10	1.42	0.27	0.83	0.05	0.41	0.02	0.64	0.08
Lemna minor	0.73	0.20	0.54	0.01	0.25	0.01	0.89	0.07	0.94	0.05
Iris wilsonii	0.76	0.06	0.66	0.12	0.34	0.09	0.85	0.06	0.92	0.04
Zizania latifolia	0.34	0.11	0.96	0.14	0.75	0.12	0.38	0.06	0.45	0.16
Sparganium stoloniferum	0.37	0.08	1.37	0.03	1.21	0.09	0.12	0.11	0.22	0.15
Typha orientalis	0.32	0.04	0.59	0.03	0.25	0.06	0.61	0.03	0.84	0.02
Phragmites australis	0.21	0.04	0.46	0.18	0.30	0.13	0.64	0.13	0.77	0.09
