# Supplementary material for: Plant Litter Submergence Affects the Water Quality of a Constructed Wetland
Source: PLoS One. 2017 Jan 27;12(1):e0171019. doi: 10.1371/journal.pone.0171019 (PMC5271387; doi:10.1371/journal.pone.0171019)
Supplement: S4 Table — (PDF) [file pone.0171019.s005.pdf]

S4 Table Main dataset for the analyses.

Time	Substrate	Species	GF	Code	Temp	Cond	TDS	Sal	ODO	Turbidity	NO3	PO4	NH4	TP	TN
14	B	1	F	B11	8.546	439.2	410	0.31	5.73	0.38	1.44	1.32	0.07	0.48	3.22
14	B	2	F	B21	8.589	449.8	419	0.32	6.73	-1.93	0.36	0.87	6.04	1.22	1.00
14	B	3	E	B31	8.584	694.5	648	0.50	1.10	3.54	0.21	1.79	0.25	0.64	6.59
14	B	4	E	B41	8.768	525.7	488	0.37	1.34	-0.97	0.30	1.10	0.61	0.29	2.85
14	B	5	E	B51	8.712	565	525	0.40	3.37	-2.73	0.34	0.92	0.30	1.62	4.03
14	B	6	E	B61	8.753	510.2	473	0.36	2.53	-3.09	0.42	0.41	0.17	0.08	1.24
14	B	7	E	B71	8.972	455.2	420	0.32	1.51	-1.51	0.66	0.10	0.39	0.02	1.57
14	B	8	CK	B81	9.05	310.8	286	0.22	11.73	-3.57	0.44	0.08	0.11	0.09	2.09
14	B	1	F	B12	8.889	417.7	386	0.29	7.71	-2.46	0.44	0.98	0.11	1.19	2.31
14	B	2	F	B22	9.077	467.2	430	0.33	6.50	-3.12	0.31	0.90	6.56	1.01	1.21
14	B	3	E	B32	8.695	634.4	590	0.46	1.03	3.13	0.26	1.18	0.98	0.38	2.07
14	B	4	E	B42	8.95	504.1	465	0.36	2.07	-2.96	0.31	0.76	0.16	0.25	2.19
14	B	5	E	B52	9.21	600.1	550	0.42	7.04	-3.12	0.37	0.92	0.14	1.31	1.80
14	B	6	E	B62	9.384	528.7	482	0.37	2.82	-3.23	0.43	0.94	0.21	1.56	1.90
14	B	7	E	B72	9.472	450.8	410	0.31	3.73	-3.10	0.41	0.00	0.23	-0.06	1.40
14	B	8	CK	B82	9.169	311.4	286	0.22	12.01	-3.56	0.45	0.09	0.21	0.08	0.91
14	B	1	F	B13	9.027	433	399	0.30	7.32	1.97	0.39	1.26	0.26	0.32	2.10
14	B	2	F	B23	8.806	357.3	331	0.25	13.19	-2.64	0.45	0.97	0.91	0.47	6.17
14	B	3	E	B33	9.138	593.9	545	0.42	2.59	0.16	0.26	1.06	0.78	0.30	2.53
14	B	4	E	B43	9.129	416.2	382	0.29	7.78	-3.39	0.38	1.27	0.23	0.61	5.08
14	B	5	E	B53	9.165	515.7	473	0.36	3.42	-2.07	0.75	0.95	0.23	1.07	2.32
14	B	6	E	B63	9.192	549.8	504	0.39	3.43	-1.43	0.41	0.07	0.24	0.00	1.42
14	B	7	E	B73	9.33	475.1	434	0.33	1.24	3.00	0.39	0.14	0.45	0.00	1.94
14	B	8	CK	B83	9.253	314.2	288	0.22	11.48	-3.56	0.39	0.24	0.25	0.08	0.84
14	A	1	F	A11	9.304	456.2	417	0.32	5.97	0.43	1.77	1.50	0.05	0.50	2.52
14	A	2	F	A21	9.254	476.2	436	0.33	4.24	0.34	0.45	1.30	3.17	0.64	7.69
14	A	3	E	A31	9.279	710.5	650	0.50	1.15	12.95	0.31	1.66	0.60	0.68	2.49
14	A	4	E	A41	9.418	502.2	458	0.35	3.74	-1.18	0.33	0.52	0.20	1.02	4.34
14	A	5	E	A51	9.448	562.8	512	0.39	7.16	0.26	0.40	0.39	0.15	1.64	2.19
14	A	6	E	A61	9.674	550.4	498	0.38	2.04	-1.28	0.40	0.57	0.52	-0.07	1.37

14	A	7	E	A71	9.462	478.8	436	0.33	1.63	5.36	0.40	0.20	0.41	0.34	1.57
14	A	8	CK	A81	9.424	309.3	282	0.21	11.26	-2.86	0.40	0.01	0.20	-0.01	0.89
14	A	1	F	A12	9.575	442.5	401	0.31	5.87	-1.42	0.84	1.41	0.20	0.51	2.71
14	A	2	F	A22	9.496	497.1	452	0.35	4.88	-1.74	0.47	0.35	7.21	1.58	8.94
14	A	3	E	A32	9.494	677.3	616	0.48	1.32	8.30	0.40	1.66	0.88	0.70	2.89
14	A	4	E	A42	9.592	422.4	383	0.29	5.52	-2.48	0.36	0.46	0.17	0.13	1.45
14	A	5	E	A52	9.648	577.5	523	0.40	7.83	3.77	0.39	0.21	0.24	0.86	1.29
14	A	6	E	A62	9.775	577.9	521	0.40	3.48	8.32	0.39	0.17	0.42	0.05	1.05
14	A	7	E	A72	9.808	470.7	424	0.32	1.44	4.81	0.41	0.27	0.94	0.17	2.39
14	A	8	CK	A82	9.771	312.5	282	0.21	10.65	-3.10	0.43	0.10	0.10	0.13	0.78
14	A	1	F	A13	9.509	430.4	391	0.30	6.83	2.40	0.50	1.09	0.11	0.49	1.18
14	A	2	F	A23	9.597	464	421	0.32	8.41	-2.83	0.48	0.20	5.04	1.08	5.80
14	A	3	E	A33	9.749	753.6	681	0.53	1.62	9.08	0.37	1.54	0.38	0.50	2.40
14	A	4	E	A43	9.732	470.1	425	0.33	5.54	-3.00	0.38	0.42	0.26	0.22	1.57
14	A	5	E	A53	9.738	567.5	513	0.39	8.77	-3.29	0.41	0.19	0.27	1.02	1.06
14	A	6	E	A63	9.739	487.5	440	0.34	3.31	-2.15	0.40	0.02	0.21	-0.04	1.04
14	A	7	E	A73	9.772	470.5	425	0.33	1.43	2.92	0.40	-0.01	0.25	-0.05	1.41
14	A	8	CK	A83	9.667	312.2	283	0.21	11.23	-2.97	0.38	0.05	0.07	-0.05	0.92
14	C	1	F	C11	9.612	404.5	367	0.28	3.54	1.67	0.46	1.33	0.78	0.62	1.39
14	C	2	F	C21	10.075	506.7	454	0.35	3.85	-2.23	0.51	0.94	11.69	5.58	12.06
14	C	3	E	C31	9.904	554.6	499	0.38	1.15	6.03	0.44	1.90	1.02	0.69	3.10
14	C	4	E	C41	9.841	456.6	411	0.31	5.87	-2.90	0.46	1.02	0.23	0.09	1.35
14	C	5	E	C51	9.957	498.8	448	0.34	6.44	0.16	0.48	0.92	0.69	1.09	1.69
14	C	6	E	C61	9.965	595.1	534	0.41	3.46	-1.12	0.38	1.38	0.86	0.19	3.21
14	C	7	E	C71	10.019	450.7	404	0.31	1.23	1.18	0.42	1.07	0.61	0.54	1.88
14	C	8	CK	C81	9.819	313.2	282	0.21	10.63	-3.80	0.37	0.67	0.27	0.13	1.10
14	C	1	F	C12	9.628	427	387	0.30	2.45	-1.52	0.47	1.60	0.89	0.55	1.87
14	C	2	F	C22	9.805	481.2	434	0.33	4.23	-0.80	0.48	0.92	11.08	1.52	12.43
14	C	3	E	C32	9.893	554.9	499	0.38	1.49	-1.72	0.89	0.74	0.14	0.54	1.93
14	C	4	E	C42	10.014	475.3	426	0.33	2.24	-1.69	0.53	1.14	0.22	0.14	1.62
14	C	5	E	C52	10.027	466	418	0.32	6.30	-1.97	0.49	0.91	0.48	1.26	1.64
14	C	6	E	C62	10.107	533.1	477	0.37	2.12	-1.44	0.40	0.99	0.20	0.09	2.02
14	C	7	E	C72	10.234	419.1	374	0.28	1.54	-1.14	0.41	0.65	0.15	0.01	1.30

14	C	8	CK	C82	9.793	311.5	281	0.21	10.89	-3.75	0.38	0.74	0.22	0.30	0.91
14	C	1	F	C13	9.627	383.4	347	0.26	8.06	62.04	0.64	1.37	1.30	0.51	2.24
14	C	2	F	C23	9.78	495.9	447	0.34	2.61	1.09	0.45	0.93	11.02	1.55	12.06
14	C	3	E	C33	9.949	495.3	445	0.34	1.60	-2.52	0.53	0.78	0.03	0.96	1.42
14	C	4	E	C43	9.986	430.2	386	0.29	7.17	-3.34	0.47	1.13	0.28	0.11	1.24
14	C	5	E	C53	10.178	485.1	433	0.33	1.83	-1.43	0.42	0.88	1.27	1.19	2.60
14	C	6	E	C63	10.222	545.4	486	0.37	1.45	-2.64	0.42	1.72	0.27	0.40	4.55
14	C	7	E	C73	10.188	426.5	381	0.29	1.30	2.65	0.38	0.92	0.18	0.05	1.42
14	C	8	CK	C83	9.833	313.8	283	0.21	11.25	-3.75	0.38	0.99	0.22	0.17	12.74
43	B	1	F	B11	14.28	486.9	392	0.30	14.52	-2.98	0.47	0.79	0.20	0.11	1.24
43	B	2	F	B21	13.82	491	400	0.31	16.92	-0.66	0.49	3.54	4.52	1.60	5.12
43	B	3	E	B31	13.352	828.7	682	0.53	1.67	6.35	0.45	1.13	0.75	0.36	3.34
43	B	4	E	B41	13.681	618.8	505	0.39	2.14	-1.73	0.40	0.30	0.56	-0.07	2.46
43	B	5	E	B51	13.808	668.6	544	0.42	9.33	-3.52	0.43	4.96	0.18	2.25	1.18
43	B	6	E	B61	13.711	592.2	483	0.37	9.32	-3.65	0.44	0.30	0.11	-0.10	1.06
43	B	7	E	B71	13.459	520.8	428	0.33	13.14	-3.35	0.42	0.13	0.14	-0.24	1.26
43	B	8	CK	B81	13.81	347.1	283	0.21	12.15	-3.75	0.42	0.10	0.11	-0.24	0.98
43	B	1	F	B12	13.941	467	379	0.29	13.34	-2.84	0.42	0.67	0.11	-0.03	0.76
43	B	2	F	B22	13.839	488.9	398	0.30	19.59	-1.21	0.46	3.72	4.98	0.90	4.71
43	B	3	E	B32	13.737	757.8	618	0.48	1.02	0.13	0.44	0.56	1.31	0.10	2.77
43	B	4	E	B42	13.495	587.9	482	0.37	3.34	-2.49	0.45	0.18	0.28	-0.12	1.97
43	B	5	E	B52	13.888	711.4	578	0.45	7.00	-3.17	0.46	5.91	0.12	2.90	1.29
43	B	6	E	B62	13.519	613.2	503	0.39	11.28	-3.28	0.48	0.43	0.12	0.07	4.57
43	B	7	E	B72	12.753	500.7	418	0.32	11.45	-3.37	0.47	0.15	0.10	0.12	0.99
43	B	8	CK	B82	13.463	334.5	275	0.21	12.51	-3.68	0.47	0.12	0.13	-0.07	0.00
43	B	1	F	B13	13.685	471.8	385	0.29	13.24	-2.19	0.49	0.85	0.18	0.24	5.08
43	B	2	F	B23	13.472	385.6	316	0.24	17.74	-2.98	0.47	0.54	0.18	0.09	4.79
43	B	3	E	B33	14.123	716.5	579	0.45	3.25	-1.94	0.46	0.31	0.71	0.06	5.47
43	B	4	E	B43	13.872	485.3	394	0.30	9.39	-3.47	0.49	1.37	0.19	0.67	3.20
43	B	5	E	B53	14.077	603.7	488	0.38	10.56	-3.50	0.49	5.48	0.14	3.62	2.74
43	B	6	E	B63	13.453	635	521	0.40	10.66	18.80	0.46	0.46	0.16	0.08	2.63
43	B	7	E	B73	13.17	524.1	433	0.33	11.20	-2.57	0.44	0.15	0.16	-0.09	6.29
43	B	8	CK	B83	13.382	341.9	281	0.21	12.33	-1.90	0.49	0.14	0.14	-0.07	2.55

43	A	1	F	A11	13.69	475.4	388	0.30	12.62	-3.48	0.51	1.17	0.15	0.20	1.20
43	A	2	F	A21	13.796	451.5	368	0.28	21.59	-2.64	2.60	1.47	0.31	0.72	5.65
43	A	3	E	A31	13.874	832.6	677	0.53	2.35	2.22	0.54	0.71	1.23	0.12	4.49
43	A	4	E	A41	13.877	585.5	476	0.37	6.63	-3.59	0.49	0.92	0.23	0.23	2.57
43	A	5	E	A51	13.839	656.9	534	0.41	8.38	5.78	0.49	4.54	0.14	2.54	1.44
43	A	6	E	A61	12.81	619	516	0.40	12.04	-2.02	0.48	0.22	0.23	-0.17	2.13
43	A	7	E	A71	12.747	525.4	439	0.34	11.75	-1.91	0.50	0.15	0.08	-0.21	2.17
43	A	8	CK	A81	12.844	335.9	280	0.21	12.42	-3.73	0.46	0.12	0.11	-0.16	1.02
43	A	1	F	A12	13.362	468.2	385	0.29	13.62	-3.77	0.47	1.00	0.14	0.21	1.69
43	A	2	F	A22	13.638	459.5	376	0.29	21.61	-2.74	1.60	1.43	1.19	0.43	4.60
43	A	3	E	A32	13.849	782	636	0.49	3.64	13.79	0.56	0.30	0.32	-0.08	1.92
43	A	4	E	A42	13.274	475.1	392	0.30	10.33	-2.22	0.48	0.63	0.79	0.14	2.82
43	A	5	E	A52	13.46	672.8	552	0.43	8.33	-3.32	0.52	3.26	0.17	1.31	1.66
43	A	6	E	A62	12.749	650.4	543	0.42	11.08	-3.71	0.42	0.48	0.16	-0.10	1.24
43	A	7	E	A72	12.6	490.3	411	0.31	15.73	0.83	0.34	0.17	0.20	-0.03	2.61
43	A	8	CK	A82	12.344	331	279	0.21	12.81	-3.58	0.36	0.13	0.15	-0.24	1.03
43	A	1	F	A13	13.192	459.1	379	0.29	12.54	-2.78	0.39	0.74	0.19	0.01	1.00
43	A	2	F	A23	13.44	418.7	344	0.26	20.36	-3.22	0.85	0.71	0.19	0.01	2.51
43	A	3	E	A33	13.606	858	702	0.55	3.42	7.90	0.34	0.50	0.77	0.06	3.56
43	A	4	E	A43	13.635	534.1	437	0.33	9.24	-2.59	0.31	0.16	0.20	-0.17	1.65
43	A	5	E	A53	13.128	664.4	550	0.43	8.61	7.55	0.38	4.24	0.10	1.38	1.14
43	A	6	E	A63	12.529	532.9	448	0.34	11.95	-3.61	0.50	0.32	0.15	-0.06	1.12
43	A	7	E	A73	12.441	484	407	0.31	16.80	1.49	0.46	0.16	0.13	-0.23	1.89
43	A	8	CK	A83	11.923	326.7	279	0.21	13.02	-3.74	0.48	0.16	0.12	-0.16	1.25
43	C	1	F	C11	13.485	393.5	323	0.25	14.26	-3.66	0.49	0.46	0.12	0.20	2.41
43	C	2	F	C21	13.366	473	389	0.30	19.37	-1.05	0.49	3.56	6.79	1.55	12.13
43	C	3	E	C31	12.58	617.9	518	0.40	5.24	-0.57	0.50	0.76	0.74	0.44	8.82
43	C	4	E	C41	11.829	498.8	426	0.33	10.59	-2.37	0.62	0.16	0.23	0.01	5.33
43	C	5	E	C51	12.515	543.9	457	0.35	13.10	-1.92	0.49	3.98	0.09	2.01	3.56
43	C	6	E	C61	12.072	658.1	559	0.43	12.11	-2.23	0.51	0.65	0.12	0.32	2.09
43	C	7	E	C71	11.788	475.5	407	0.31	11.64	-2.87	0.52	0.19	0.16	0.02	3.30
43	C	8	CK	C81	11.73	326	279	0.21	12.30	-3.81	0.49	0.12	0.08	0.01	0.80
43	C	1	F	C12	12.682	407.6	341	0.26	13.67	-2.52	0.51	0.64	0.27	0.34	1.90

43	C	2	F	C22	12.774	428.1	357	0.27	21.02	-1.22	0.41	1.74	5.12	1.07	8.58
43	C	3	E	C32	12.488	613.6	516	0.40	10.80	-2.65	0.37	3.30	0.47	1.34	2.25
43	C	4	E	C42	12.081	517.8	440	0.34	11.40	-3.18	0.39	0.30	0.28	0.15	2.70
43	C	5	E	C52	12.299	508	429	0.33	11.84	-3.58	0.39	3.88	0.24	2.00	1.65
43	C	6	E	C62	12.019	578.3	492	0.38	9.41	-2.42	0.36	0.42	0.17	0.13	1.93
43	C	7	E	C72	11.682	445.1	382	0.29	9.25	-1.52	0.32	0.15	0.25	-0.02	3.02
43	C	8	CK	C82	11.168	324	282	0.21	11.87	-3.85	0.45	0.13	0.14	-0.10	0.62
43	C	1	F	C13	13.056	364.3	302	0.23	12.88	-1.01	0.47	0.39	0.46	0.17	1.60
43	C	2	F	C23	12.698	420.2	351	0.27	21.37	-0.48	0.46	1.63	4.71	1.13	10.08
43	C	3	E	C33	12.636	543.8	456	0.35	10.68	-2.42	0.45	1.62	0.55	1.11	2.32
43	C	4	E	C43	12.199	478.2	405	0.31	9.93	-3.68	0.47	0.17	0.18	0.05	2.31
43	C	5	E	C53	12.167	503.8	427	0.33	12.17	-2.43	0.45	3.46	0.10	2.40	1.59
43	C	6	E	C63	11.813	574.3	491	0.38	10.92	-2.61	0.47	1.27	0.20	0.82	3.17
43	C	7	E	C73	11.54	447.6	386	0.29	7.28	-2.85	0.47	0.18	0.17	-0.04	1.31
43	C	8	CK	C83	11.239	318.6	277	0.21	12.15	-3.82	0.48	0.14	0.13	0.04	0.81
28	B	1	F	B11	1.852	372.9	428	0.32	12.90	2.27	0.80	1.11	0.89	1.11	3.24
28	B	2	F	B21	0.965	374.2	443	0.33	14.52	0.07	0.42	4.34	12.30	3.47	9.46
28	B	3	E	B31	1.245	576.9	676	0.52	2.66	20.59	0.38	1.82	0.78	2.23	3.51
28	B	4	E	B41	1.377	441.5	515	0.39	1.72	-0.90	0.41	0.56	0.12	0.74	2.38
28	B	5	E	B51	2.378	488	550	0.42	4.86	-3.17	0.39	3.43	0.09	2.64	1.32
28	B	6	E	B61	2.419	430.5	484	0.37	5.80	-1.39	0.42	0.21	0.09	0.16	1.25
28	B	7	E	B71	2.817	383.5	426	0.32	4.80	-3.21	0.43	0.16	0.10	0.17	1.60
28	B	8	CK	B81	3.257	253.3	277	0.21	12.33	-3.64	0.42	0.12	0.09	0.27	0.77
28	B	1	F	B12	1.777	334.5	385	0.29	9.52	-1.70	0.43	0.71	0.11	0.88	1.17
28	B	2	F	B22	1.924	381.7	437	0.33	15.70	-2.70	0.42	3.23	8.15	1.81	7.07
28	B	3	E	B32	1.805	533.3	613	0.47	2.51	6.54	0.51	0.89	1.33	0.75	2.35
28	B	4	E	B42	2.433	424	477	0.36	2.35	-2.79	0.47	0.60	0.22	0.90	2.03
28	B	5	E	B52	3.079	496.5	547	0.42	4.32	-2.36	0.47	4.34	0.12	4.22	1.54
28	B	6	E	B62	3.245	456.7	500	0.38	7.67	-3.50	0.59	0.26	0.12	0.50	1.05
28	B	7	E	B72	3.973	384.2	411	0.31	8.13	-3.10	0.47	0.13	0.09	0.20	1.36
28	B	8	CK	B82	4.246	260.4	276	0.21	12.91	-3.62	0.47	0.12	0.09	0.11	0.83
28	B	1	F	B13	2.669	356.5	398	0.30	10.05	-1.93	0.48	1.32	0.22	1.12	0.96
28	B	2	F	B23	2.561	302.3	339	0.25	12.81	-2.58	0.44	0.68	0.14	0.84	1.82

28	B	3	E	B33	2.902	503.2	557	0.43	2.23	0.44	0.48	0.44	0.09	0.69	1.71
28	B	4	E	B43	2.97	354.2	391	0.30	7.46	-3.51	0.49	1.33	0.11	3.24	1.62
28	B	5	E	B53	4.312	454.3	481	0.37	7.63	-3.38	0.45	4.14	0.10	2.82	1.30
28	B	6	E	B63	4.589	482.9	506	0.39	8.57	-3.33	0.47	0.27	0.09	0.25	1.33
28	B	7	E	B73	5.236	422.8	435	0.33	4.97	-2.61	0.46	0.15	0.11	0.29	2.27
28	B	8	CK	B83	5.048	274	283	0.21	12.68	-3.65	0.42	0.13	0.14	0.16	0.95
28	A	1	F	A11	4.278	377.3	400	0.30	15.20	-3.36	0.63	1.63	0.11	2.10	3.60
28	A	2	F	A21	4.451	414	436	0.33	15.44	-3.32	0.76	4.11	6.31	6.43	9.97
28	A	3	E	A31	4.58	630.1	661	0.51	2.63	46.96	0.53	0.91	0.39	1.27	3.03
28	A	4	E	A41	5.119	453	467	0.36	5.94	-2.80	0.49	0.94	0.12	0.98	2.44
28	A	5	E	A51	5.534	509.9	519	0.40	8.04	-2.79	0.44	4.01	0.10	3.82	1.65
28	A	6	E	A61	5.957	499.5	502	0.38	5.87	-2.50	0.41	0.18	0.14	0.52	1.51
28	A	7	E	A71	5.823	434.9	439	0.33	4.98	-0.31	0.41	0.13	0.17	0.25	2.21
28	A	8	CK	A81	5.682	277.4	281	0.21	12.05	-3.68	0.45	0.10	0.11	0.09	0.86
28	A	1	F	A12	5.271	387	397	0.30	13.20	-2.85	0.44	0.88	0.12	1.16	1.09
28	A	2	F	A22	5.559	435.9	444	0.34	16.17	-2.40	0.63	3.92	6.53	2.54	9.74
28	A	3	E	A32	5.866	624.9	630	0.49	2.82	14.23	0.45	1.24	0.48	1.01	3.42
28	A	4	E	A42	5.613	382.2	388	0.29	8.34	-3.74	0.43	0.24	0.12	0.33	1.56
28	A	5	E	A52	6.452	542.1	537	0.41	6.70	-3.61	0.47	3.85	0.11	2.46	1.49
28	A	6	E	A62	6.54	536.3	530	0.41	10.51	-3.37	0.46	0.18	0.11	0.25	1.02
28	A	7	E	A72	6.962	428.5	418	0.32	6.38	-1.59	0.42	0.13	0.27	0.23	1.97
28	A	8	CK	A82	6.008	280.2	281	0.21	12.27	-3.74	0.46	0.12	0.12	0.08	0.90
28	A	1	F	A13	5.801	381.5	386	0.29	12.86	4.10	0.48	0.76	0.12	0.83	1.06
28	A	2	F	A23	6.318	393	391	0.30	16.38	-3.04	1.05	1.50	3.26	1.60	4.49
28	A	3	E	A33	6.555	688.6	680	0.53	3.72	22.39	0.53	0.97	0.31	1.02	2.60
28	A	4	E	A43	6.605	437.8	432	0.33	7.65	-3.63	0.50	0.22	0.14	0.16	1.73
28	A	5	E	A53	6.785	538.8	529	0.41	9.59	-3.63	0.46	4.31	0.12	2.78	1.09
28	A	6	E	A63	6.728	450.6	443	0.34	10.97	-3.46	0.41	0.32	0.14	0.15	1.14
28	A	7	E	A73	7.223	440	426	0.33	7.44	-1.12	0.55	0.12	0.24	0.17	1.75
28	A	8	CK	A83	6.368	282.3	280	0.21	12.28	-3.67	0.43	0.11	0.12	0.14	0.91
28	C	1	F	C11	6.481	352.5	349	0.26	15.76	-1.42	0.52	1.08	0.14	1.21	1.08
28	C	2	F	C21	7.159	473.9	460	0.35	9.06	-3.29	0.49	4.31	12.63	3.93	9.16
28	C	3	E	C31	7.066	519.8	506	0.39	2.72	6.27	0.48	0.85	0.57	1.00	1.72

28	C	4	E	C41	6.867	429.7	421	0.32	9.69	-3.30	0.40	0.28	0.15	0.70	1.63
28	C	5	E	C51	6.962	468.9	458	0.35	10.41	-1.54	0.57	3.65	0.13	2.42	1.42
28	C	6	E	C61	7.259	568.1	550	0.42	10.07	-1.34	0.46	0.91	0.12	1.06	0.95
28	C	7	E	C71	7.559	421.9	405	0.31	4.11	-1.75	0.50	0.27	0.09	0.37	1.58
28	C	8	CK	C81	6.431	285.1	283	0.21	12.42	-3.74	0.49	0.08	0.15	0.09	0.69
28	C	1	F	C12	6.992	376.2	367	0.28	15.30	-3.04	0.47	1.06	0.12	1.30	0.83
28	C	2	F	C22	7.305	451.5	436	0.33	11.56	-3.30	0.48	3.79	11.16	2.45	8.45
28	C	3	E	C32	7.736	536.9	513	0.39	5.20	-1.69	0.38	3.30	0.48	2.28	1.30
28	C	4	E	C42	7.551	452	434	0.33	6.01	-2.89	0.35	1.42	0.11	0.55	1.13
28	C	5	E	C52	7.329	440.6	426	0.33	9.50	-2.63	0.44	3.56	0.10	1.70	0.94
28	C	6	E	C62	7.902	396	376	0.29	7.01	-2.00	0.43	0.37	0.11	1.40	0.97
28	C	7	E	C72							0.43	0.12	0.14	0.12	1.43
28	C	8	CK	C82	6.569	281.9	278	0.21	12.75	-3.61	0.41	0.08	0.08	0.01	0.64
28	C	1	F	C13	7.472	342.7	330	0.25	15.37	-2.99	0.41	0.40	0.09	0.22	0.75
28	C	2	F	C23	7.607	469.8	450	0.34	11.33	-2.76	0.49	3.98	13.05	2.30	11.12
28	C	3	E	C33	7.869	459.1	437	0.33	7.27	-1.71	0.46	1.60	0.15	1.09	0.89
28	C	4	E	C43	7.319	408.8	395	0.30	8.48	-3.48	0.44	0.17	0.10	-0.08	1.09
28	C	5	E	C53	7.75	438.5	419	0.32	16.59	-3.08	0.49	2.94	0.11	1.74	0.89
28	C	6	E	C63	7.959	512.9	487	0.37	10.34	-2.78	0.47	1.60	0.14	0.81	1.18
28	C	7	E	C73	7.929	400.9	381	0.29	4.05	-1.47	0.47	0.17	0.11	0.17	1.05
28	C	8	CK	C83	6.659	281.3	277	0.21	12.88	-3.73	0.44	0.12	0.09	-0.21	0.59
57	B	1	F	B11	5.708	363.5	368	0.28	14.53	-3.66	0.24	0.64	0.15	0.14	1.37
57	B	2	F	B21	5.442	317	324	0.24	24.06	-0.94	0.35	0.55	0.12	0.76	3.09
57	B	3	E	B31	5.329	658.9	675	0.52	6.39	4.68	0.30	0.26	0.57	1.59	3.27
57	B	4	E	B41	5.536	505.5	515	0.39	5.24	-1.96	0.37	-0.04	0.30	0.05	3.41
57	B	5	E	B51	6.188	537.2	537	0.41	10.97	-3.08	0.36	0.22	0.11	0.88	1.55
57	B	6	E	B61	6.346	479.4	477	0.36	11.92	-3.57	0.34	0.25	0.21	0.05	1.29
57	B	7	E	B71	6.403	391.4	388	0.30	13.52	-3.42	0.35	0.14	0.24	-0.13	1.65
57	B	8	CK	B81	6.198	279.3	279	0.21	13.23	-3.71	0.32	0.01	0.15	-0.15	0.80
57	B	1	F	B12	6.507	363	359	0.27	13.92	2.88	0.37	0.63	0.15	0.04	1.14
57	B	2	F	B22	6.235	357.7	357	0.27	20.02	-0.95	0.35	0.45	0.11	1.32	3.27
57	B	3	E	B32	6.423	619.9	615	0.47	5.54	0.42	0.35	0.09	0.44	-0.07	3.22
57	B	4	E	B42	6.122	486	486	0.37	9.35	-2.62	0.39	-0.05	0.20	0.04	3.10

57	B	5	E	B52	6.7	583.6	574	0.44	9.60	-1.05	0.38	0.34	0.22	0.15	2.55
57	B	6	E	B62	6.767	512.4	503	0.39	11.92	-3.68	0.44	0.33	0.15	0.19	2.35
57	B	7	E	B72	6.468	414.8	411	0.31	12.37	-2.75	0.43	0.06	0.17	0.05	2.22
57	B	8	CK	B82	6.534	273.1	270	0.20	13.09	-3.71	0.38	-0.03	0.13	0.02	1.59
57	B	1	F	B13	6.725	371.7	365	0.28	13.73	-3.59	0.41	0.68	0.15	0.23	1.70
57	B	2	F	B23	6.408	275.5	273	0.21	16.91	-2.78	0.39	0.15	0.12	0.03	2.82
57	B	3	E	B33	6.925	586	573	0.44	9.32	-2.34	0.36	0.01	0.37	-0.01	2.73
57	B	4	E	B43	6.733	397.8	391	0.30	11.04	-3.57	0.41	1.05	0.18	0.41	2.05
57	B	5	E	B53	6.782	493	484	0.37	11.69	5.78	0.38	0.35	0.17	1.16	1.55
57	B	6	E	B63	6.846	525.6	515	0.40	12.23	-2.85	0.40	0.31	0.16	0.05	1.35
57	B	7	E	B73	6.751	401.1	394	0.30	14.86	-2.71	0.41	0.03	0.17	0.26	2.40
57	B	8	CK	B83	6.738	281.6	277	0.21	13.46	-3.70	0.39	0.35	0.14	-0.03	1.23
57	A	1	F	A11	7.147	382.6	372	0.28	13.53	-3.60	0.38	0.88	0.16	0.31	3.12
57	A	2	F	A21	7.237	365.7	354	0.27	16.75	-0.45	0.41	0.31	0.15	0.18	8.36
57	A	3	E	A31	7.49	678	652	0.51	9.41	0.51	0.40	-0.02	0.26	0.06	4.48
57	A	4	E	A41	6.808	478.8	470	0.36	9.69	-1.87	0.44	0.56	0.24	0.14	3.25
57	A	5	E	A51	7.108	550.2	535	0.41	10.61	-2.75	0.40	0.32	0.19	1.35	1.87
57	A	6	E	A61	7.021	524.8	512	0.39	11.61	-3.57	0.34	0.26	0.16	-0.02	1.63
57	A	7	E	A71	7.214	427	414	0.32	13.82	-1.29	0.35	0.34	0.22	0.04	1.86
57	A	8	CK	A81	6.926	281.6	275	0.21	13.36	-3.52	0.39	-0.02	0.16	-0.08	0.88
57	A	1	F	A12	7.492	383.7	369	0.28	12.99	-3.67	0.38	0.53	0.15	0.33	1.01
57	A	2	F	A22	7.882	378.9	360	0.27	16.63	0.37	0.38	0.38	0.16	0.16	3.20
57	A	3	E	A32	7.766	652.8	623	0.48	5.58	5.32	0.40	0.07	0.36	0.07	2.58
57	A	4	E	A42	6.805	395.9	388	0.30	11.61	-3.74	0.39	-0.02	0.14	-0.15	1.68
57	A	5	E	A52	7.352	569.7	550	0.42	11.40	-3.72	0.40	0.19	0.16	0.98	1.60
57	A	6	E	A62	7.304	599	579	0.45	11.44	-3.63	0.42	0.18	0.19	-0.10	2.16
57	A	7	E	A72	7.623	361.3	346	0.26	15.78	3.34	0.40	0.06	0.24	0.00	3.66
57	A	8	CK	A82	7.073	283.9	276	0.21	13.78	-3.47	0.38	0.01	0.14	-0.08	1.04
57	A	1	F	A13	7.901	381.1	362	0.28	12.98	-3.76	0.45	0.45	0.19	0.12	1.40
57	A	2	F	A23	8.167	341	322	0.24	15.75	-0.78	0.44	0.31	0.14	0.05	3.53
57	A	3	E	A33	8.023	732.4	694	0.54	12.45	-0.80	0.43	0.00	0.20	0.16	2.38
57	A	4	E	A43	7.954	462.8	439	0.34	11.31	-3.67	0.42	0.38	0.19	0.30	1.31
57	A	5	E	A53	7.99	580.9	551	0.42	11.21	-3.73	0.39	0.24	0.03	1.14	1.16

57	A	6	E	A63	7.922	473.2	449	0.34	11.32	-2.96	0.32	0.14	0.17	0.02	0.96
57	A	7	E	A73	7.664	372.7	357	0.27	15.09	2.42	0.46	0.02	0.23	0.00	1.98
57	A	8	CK	A83	7.409	283.9	274	0.21	13.60	-3.63	0.39	0.02	0.19	-0.08	0.88
57	C	1	F	C11	8.279	327.3	308	0.23	13.23	-1.20	0.45	0.17	0.16	0.06	1.31
57	C	2	F	C21	8.473	339.9	318	0.24	18.79	0.24	0.44	0.64	1.14	0.20	5.82
57	C	3	E	C31	7.851	533.9	508	0.39	13.99	-2.80	0.41	0.42	0.29	0.08	2.18
57	C	4	E	C41	7.787	448.4	428	0.33	12.76	-3.39	0.33	-0.06	0.13	-0.11	1.51
57	C	5	E	C51	8.029	488.4	463	0.35	13.27	-3.40	0.49	1.71	0.13	0.47	1.28
57	C	6	E	C61	8.083	580.5	549	0.42	13.46	-3.18	0.41	0.27	0.11	-0.03	0.99
57	C	7	E	C71	7.864	406.1	386	0.29	15.06	-3.56	0.43	-0.06	0.09	-0.06	1.54
57	C	8	CK	C81	7.59	293.4	281	0.21	13.42	-3.76	0.44	0.08	0.13	0.38	0.72
57	C	1	F	C12	8.452	379	355	0.27	20.97	15.99	0.38	0.43	0.13	0.13	1.18
57	C	2	F	C22	8.203	503	474	0.36	13.42	-2.29	0.42	0.17	0.14	0.06	3.64
57	C	3	E	C32	7.596	453.7	435	0.33	14.39	-3.05	0.33	1.24	0.20	0.39	1.44
57	C	4	E	C42	8.057	451.2	427	0.33	12.65	-3.62	0.29	0.11	0.12	-0.06	1.67
57	C	5	E	C52	8.23	518.5	488	0.37	13.50	-0.64	0.38	0.22	0.04	0.77	1.09
57	C	6	E	C62	7.963	376.9	358	0.27	15.70	-1.18	0.40	0.27	0.15	-0.05	1.38
57	C	7	E	C72	7.779	294.8	281	0.21	13.47	-2.94	0.37	-0.01	0.15	-0.13	0.97
57	C	8	CK	C82	7.63	294.1	282	0.21	13.20	-3.71	0.35	0.13	0.17	0.00	0.56
57	C	1	F	C13	9.238	304.4	279	0.21	13.01	-3.61	0.36	0.03	0.15	-0.03	0.68
57	C	2	F	C23	8.93	336.2	310	0.24	19.05	4.95	0.37	0.37	0.32	0.16	2.75
57	C	3	E	C33	8.704	489.7	455	0.35	12.92	-2.92	0.36	1.04	0.16	0.35	1.17
57	C	4	E	C43	8.428	441	413	0.32	10.95	-2.77	0.37	0.13	0.17	-0.05	1.23
57	C	5	E	C53	8.645	464.7	433	0.33	12.00	-3.67	0.37	1.92	0.15	6.50	1.03
57	C	6	E	C63	8.612	528.3	492	0.38	12.16	-2.89	0.37	0.87	0.17	0.42	1.59
57	C	7	E	C73	8.576	398.6	372	0.28	9.43	-2.87	0.44	0.27	0.18	0.00	1.39
57	C	8	CK	C83	7.888	290.8	276	0.21	12.86	-3.75	0.41	0.06	0.15	-0.10	0.56
